# Emerging Triglyceride-Rich Lipoprotein Targeted Therapies: An Unmet Need in Cardiometabolic Disease

**DOI:** 10.3390/pharmaceutics17091107

**Published:** 2025-08-25

**Authors:** Jorge Ferreira, Miguel Domingues, António Ferreira

**Affiliations:** Department of Cardiology, Hospital de Santa Cruz, Western Lisbon Local Health Unit, 1449-005 Lisbon, Portugal; mvdomingues@ulslo.min-saude.pt (M.D.);

**Keywords:** angiopoietin-like 3, apolipoprotein C-III, atherosclerotic cardiovascular disease, hypertriglyceridemia, triglyceride-rich lipoprotein

## Abstract

**Background/Objectives**: Hypertriglyceridemia (HTG) is a common multifactorial metabolic disorder often with genetic predisposition. Multiple lines of evidence support a causal role of triglyceride-rich lipoproteins (TRLs) in atherosclerotic cardiovascular disease (ASCVD), with severe HTG leading to pancreatitis and hepatic steatosis. This review covers TRL metabolism, causes and consequences of HTG, current management, and emerging TRL-targeted therapies. **Methods:** A narrative review was conducted. **Results:** Pharmacologic therapy with fibrates and omega-3 fatty acids remains the standard treatment for HTG but its efficacy in preventing pancreatitis and ASCVD is limited. Genetic studies have identified apolipoprotein C-III (ApoC-III) and angiopoietin-like 3 (ANGPTL3), both inhibitors of lipoprotein lipase, as potential therapeutic targets for reducing TG levels and ASCVD risk. Monoclonal antibodies and RNA-based therapies have enabled the development of inhibitors of ApoC-III and ANGPTL3, with promising results in phase 2 and small phase 3 trials. Angiopoietin-like 4 inhibitors and Fibroblast growth factor 21 analogs are in early-stage clinical development. **Conclusions:** Current pharmacologic therapies exhibit notable limitations in effectively managing severe HTG and in reducing the risk of ASCVD. Emerging therapies targeting TRLs metabolism showed favourable results in initial clinical trials.

## 1. Synthesis and Metabolism of Triglycerides

Triglycerides (TGs) are a class of lipids composed of a glycerol backbone esterified with three fatty acid molecules. The fatty acid chains may vary in length and degree of saturation, contributing to the physical and metabolic properties of the triglyceride (TG) molecule [1,2]. In animals, TG components represent the principal form of energy storage, predominantly within adipocytes of adipose tissue.

**Figure 1:**
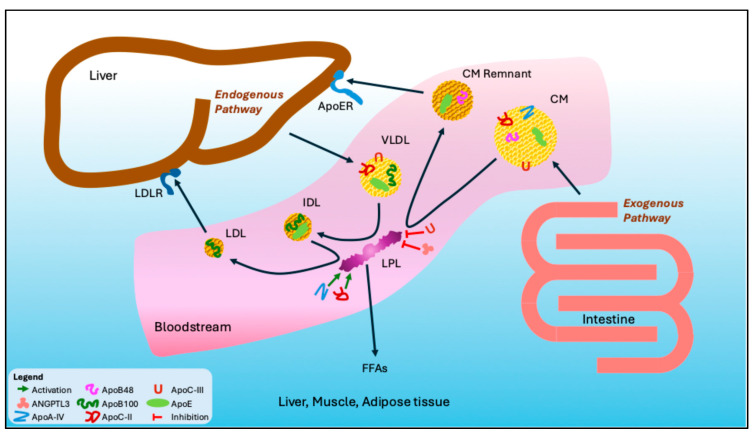
Synthesis and metabolism of triglyceride-rich lipoproteins. Lipoprotein lipase (LPL) is a key enzyme in TRLs metabolism. LPL is activated by ApoA-IV and ApoC-II and inhibited by ApoC-III and ANGPTL3. ApoER: Apolipoprotein E receptor; CM: Chylomicron; FFAs: Free fatty acids; IDL: Intermediate-density lipoprotein; LDL: Low-density lipoprotein; LDLR: Low-density lipoprotein receptor; LPL: Lipoprotein lipase; VLDL: Very low-density lipoprotein.

TGs are synthesized both in the intestine and the liver and are transported in the vascular system primarily within chylomicrons and very low-density lipoproteins (VLDL) (Figure 1) [3]. Within enterocytes, hydrolysed dietary lipids are re-esterified to form TGs and assembled into chylomicrons, the largest lipoproteins that facilitate the transport of dietary lipids via the lymphatic system into the bloodstream [4]. In addition to their high TG content, chylomicrons contain a single molecule of apolipoprotein B48 (ApoB48), which corresponds exactly to the N-terminal 48% of apoliprotein B100 (ApoB100), and smaller amounts of phospholipids, cholesterol and fat-soluble vitamins [5]. Upon entering the circulation, chylomicrons acquire apolipoprotein C (ApoC-I, C-II and C-III), apolipoprotein A (ApoA-I, II, IV and V), and apolipoprotein E (ApoE) through transfer from high-density lipoproteins (HDL), which is essential for their metabolic processing and clearance [6].

In parallel, hepatocytes synthesize TGs de novo or reassemble them from circulating free fatty acids, packaging them into VLDL particles for distribution to peripheral tissues [3]. The assembly of VLDL involves the progressive lipidation of their core structural ApoB100. VLDL particles are secreted in varying sizes and TG content, broadly classified into VLDL-1, which are larger and TG-rich, and VLDL-2, which are smaller and contain relatively less TGs [7]. In the circulation, VLDL acquire additional HDL-derived apolipoproteins, including ApoC and ApoE isoforms, modulating their interaction with lipolytic enzymes and cellular receptors [6].

Within the vascular compartment, chylomicrons and VLDL are exposed to the action of lipoprotein lipase (LPL), an enzyme located on the surface of endothelial cells in skeletal muscle, myocardium and adipose tissue (Figure 1) [3]. The chaperone protein lipase maturation factor 1 (LMF1) is essential for LPL to attain functional maturation and be secreted appropriately [8]. After being secreted by adipose and muscle cells, the enzyme is transported to the luminal surface of the capillary endothelium by glycosylphosphatidylinositol-anchored high-density lipoprotein-binding protein 1 (GPIHBP1), where it becomes attached to glycosaminoglycans [8]. LPL catalyzes the hydrolysis of TG, facilitating the release of free fatty acids for energy production or storage. The activity of LPL is increased by ApoC-II, whereas ApoC-III functions as an endogenous inhibitor [9]. Additional activators of LPL include ApoA-IV, ApoA-V, insulin and leptin while the growth factors angiopoietin-like proteins 3 (ANGPTL3), 4 (ANGPTL4) and 8 (ANGPTL8) act as inhibitors of LPL activity.

As TGs are progressively hydrolysed from these lipoproteins, they undergo a reduction in particle size, accompanied by an increase in density and a higher relative content of cholesterol [10]. Chylomicrons are progressively transformed into chylomicron remnants, which are rapidly cleared by the liver. VLDL-1 particles are converted into smaller VLDL-2 particles, which are subsequently remodelled into intermediate-density lipoproteins (IDL) and low-density lipoproteins (LDL) and removed from the circulation by hepatic LDL-receptors through interactions involving ApoB100 and ApoE (Figure 1) [3].

The metabolism of TG-rich lipoproteins (TRLs) is intricately linked to that of cholesterol-rich lipoproteins through the action of cholesteryl ester transfer protein (CETP) [11]. This plasma glycoprotein mediates the transfer of cholesteryl esters from HDL to VLDL and chylomicron remnants, while reciprocally transferring TGs from these lipoproteins to HDL, thereby playing a critical role in lipoprotein remodelling and lipid homeostasis. Finally, gut microbiome is increasingly recognized as a regulator of lipid metabolism, influencing this process through the catabolism of dietary lipids and the synthesis of new lipid molecules [12].

## 2. Causes and Clinical Relevance of Hypertriglyceridemia

Serum TG levels are classified as elevated when exceeding 150 mg/dL (1.7 mmol/L), as measured on a lipid panel obtained after a minimum of 10 to 12 h of fasting [13,14]. TG levels are considered very high above 500 mg/dL (5.6 mmol/L), and hypertriglyceridemia (HTG) is classified as severe when levels surpass 1000 mg/dL (11.3 mmol/L). HTG is a common metabolic disorder, affecting approximately 10 to 20 percent of the adult population [2,13]. Serum TG levels are generally higher in men than in women and tend to increase with age in both sexes. Approximately 2 percent of individuals exhibit TG levels above 500 mg/dL, and severe HTG is observed in less than 0.1 percent of the population [15,16].

### 2.1. Causes of Hypertriglyceridemia

HTG is typically a multifactorial condition, arising from a combination of increased synthesis, and/or impaired clearance of TRLs, often with underlying genetic predisposition [2,17,18]. It is commonly associated with high-calorie, high-fat diets, excessive alcohol consumption, physical inactivity, overweight or obesity and diabetes mellitus. Other common secondary forms of HTG include chronic kidney disease and the use of TG-raising medications (e.g., beta-blockers, clozapine, cyclosporine, glucocorticoids, HIV protease inhibitors, estrogens, propofol, sirolimus, tacrolimus, thiazides).

Primary severe HTG is driven by a complex genetic architecture comprising rare high-impact monogenic mutations and the additive effects of common polygenic variants [13,17,18]. These cases can be further exacerbated by secondary factors that amplify the phenotypic expression of TG elevations.

Approximately 2% of patients with primary severe HTG have monogenic chylomicronaemia, also known as familial chylomicronaemia syndrome (FCS, formerly classified as Type 1 hyperlipoproteinemia in the Fredrickson classification) [2,13,17,18]. This rare autosomal recessive disorder is caused by biallelic loss of function mutations—either homozygous or compound heterozygous—in genes essential for LPL activity, including LPL (80% of cases), ApoC-II, ApoA-V, LMF1, and GPIHBP1.

Most remaining cases of primary severe HTG are classified as Multifactorial HTG (MH, formerly Type 5 hyperlipoproteinemia) with a polygenic basis that includes contributions from rare heterozygous variants in the five canonical FCS genes, as well as common TG-raising variants identified in genome-wide association studies (GWAS), including the ApoC-III and ANGPTL3 genes [13,17,18].

Primary HTG with TG levels below 1000 mg/dL (11.3 mmol/L) is also polygenic in origin [2,17,18]. Combined hyperlipidaemia (previously known as Type 2B hyperlipoproteinemia) additionally carries a polygenic predisposition to elevated LDL-C. In dysbetalipoproteinemia (formerly Type 3 hyperlipoproteinemia), patients carry a further ApoE2 homozygosity or a rare binding-defective dominant mutation in ApoE, leading to impaired hepatic clearance of remnant lipoproteins. Simple HTG (formerly Type 4 hyperlipoproteinemia) is like severe HTG but with a lower genetic burden.

Finally, the previously described secondary factors of HTG may, in some patients, be sufficient to trigger its expression, but they often interact with polygenic susceptibility [2,18].

### 2.2. Clinical Relevance of Hypertriglyceridemia

HTG is a common cause of pancreatitis, with the risk increasing in a dose-dependent manner as serum TG concentrations rise [19]. The risk of acute pancreatitis increases markedly at TG levels above 1000 mg (11.3 mmol/L), where the incidence is approximately 5% [20].

Metabolic dysfunction-associated steatotic liver disease (MASLD) formerly known as non-alcoholic fatty liver disease (NAFLD), is characterized by hepatic steatosis in individuals with at least one metabolic risk factor [21]. It is particularly common in individuals with elevated TG levels, insulin resistance and obesity. MASLD has emerged as one of the most prevalent liver disorders with an estimated worldwide prevalence of approximately 30 percent. The disease spectrum ranges from simple, potentially reversible hepatic steatosis to non-alcoholic steatohepatitis (NASH), a progressive form that can lead to cirrhosis [22].

Eruptive xanthomas and lipemia retinalis may occur when TG levels exceed 2000 mg/dL (22.6 mmol/L), indicating dangerously elevated serum concentrations [23,24].

Finally, elevated levels of TRLs are increasingly recognized as a key contributor to the development of atherosclerotic cardiovascular disease (ASCVD) [25].

## 3. Triglycerides and Risk of Atherosclerotic Cardiovascular Disease

Elevated TG levels are associated with an increased risk of ASCVD, although its causal role remains uncertain. The evidence is derived from epidemiological, genetic and biological data [26].

### 3.1. Epidemiological Data

Multiple epidemiological studies have shown that elevated TG levels, both fasting and non-fasting, are associated with an increased risk of ASCVD. A meta-analysis of 17 population-based studies found that each 1 mmol/L increase in TG levels was linked to a 76% higher risk of cardiovascular disease in women and a 31% higher risk in men [27]. A more recent meta-analysis of 29 studies, involving 262,525 participants and 10,158 incident cases of coronary heart disease (CHD), showed that individuals in the highest tertile of TG levels had a significantly 72 percent higher adjusted risk of CHD compared to those in the lowest tertile [28].

Research conducted within the Danish population has served as a foundational reference for elucidating the association between TG levels and ASCVD. In the Copenhagen City Heart Study, a prospective cohort study initiated in 1976 with 7587 women and 6394 men followed for 27 years, non-fasting TG levels exceeding 440 mg/dL (>5 mmol/L) compared to <88 mg/dL (1 mmol/L) were associated with a markedly increased risk of myocardial infarction: 17-fold in women and 5-fold in men [29]. The associated risk increases were 5- and 3-fold for ischemic stroke, and 4- and 2-fold for all-cause mortality, respectively. In the Copenhagen General Population Study, which included over 100,000 individuals since 2003 and followed for up to 14 years, the absolute risk of myocardial infarction increased approximately 4-fold among those with non-fasting TG levels above 440 mg/dL (>5 mmol/L) compared to those with levels below 88 mg/dL (1 mmol/L) [26,30]. The increase in absolute risk of ischemic stroke was more modest [30].

A prospective study of 26,509 initially healthy U.S. women participating in the Women’s Health Study, conducted between 1992 and 1995, with a median follow-up of 11.4 years found that non-fasting TG levels were strongly and independently associated with cardiovascular events in fully adjusted models [31]. Hazard ratios (95% confidence intervals) across increasing tertiles were 1.0 (reference), 1.44 (0.90–2.29), and 1.98 (1.21–3.25), with a significant trend (*p* = 0.006).

The Emerging Risk Factors Collaboration analyzed data from 68 long-term prospective studies, mostly conducted in Europe and North America, involving over 300,000 individuals [32]. The analysis found that non-fasting and fasting TG levels were associated with an increased risk of CHD. After adjusting for non-lipid risk factors, the hazard ratio for CHD across TG quantiles was 1.37 (95% CI, 1.31–1.42). However, this association was attenuated to 0.99 (95% CI, 0.94–1.05) after additional adjustment for HDL-C and non-HDL-C. These findings suggest that it may not be elevated TGs alone driving the risk of CHD, but also the cholesterol content within remnant lipoprotein particles.

The remnant cholesterol hypothesis has been supported by several studies, particularly the Copenhagen studies, which demonstrated that hazard ratios for CHD increased across quintiles of non-fasting TRL cholesterol [33]. In observational analyses, the hazard ratio for individuals in the highest versus lowest quintile of remnant cholesterol was 2.3 (95% CI: 1.7–3.1).

### 3.2. Genetic Data

Genetic studies, including Mendelian randomization and GWAS, support a link between HTG and ASCVD [26,34,35].

The Global Lipids Genetics Consortium conducted a meta-analysis involving 188,578 genotyped individuals, examining 185 single-nucleotide polymorphisms (SNPs) associated with TGs, LDL-C and HDL-C [36]. The analysis revealed that genetically elevated TG levels were strongly linked to increased risk of CHD, even after adjusting for LDL-C and HDL-C levels.

Data from 17 studies involving 62,199 individuals of European origin, including 12,099 CHD events, were analyzed in a mendelian randomization meta-analysis using SNPs known to be associated with TGs, HDL-C and LDL-C [37]. For TGs, both the unrestricted allele score (67 SNPs) and the restricted allele score (27 SNPs) were significantly associated with CHD, with odds ratio per 1-log unit increase of 1.62 (95% CI: 1.24–2.11) and 1.61 (95% CI: 1.00–2.59), respectively.

A Mendelian randomization study involving 73,513 genotyped individuals from Copenhagen, with 11,984 cases of CHD recorded between 1976 and 2010, analyzed 15 genetic variants [33]. These variants were associated with non-fasting remnant cholesterol, a combination of non-fasting remnant cholesterol and HDL-C, HDL-C alone, and LDL-C alone (used as a positive control). The study found that a 39 mg/dL (1 mmol/L) increase in non-fasting remnant cholesterol was linked to a 2.8-fold higher risk of CHD, independent of reduced HDL-C levels. These findings support that elevated cholesterol content in TRL particles directly contributes to the development of CHD.

Genetic variants affecting TRL metabolism, particularly within the LPL gene and other genes that influence LPL activity, have been linked to ASCVD. In a combined analysis of the Myocardial Infarction Genetics Consortium and the Geisinger Health System DiscovEHR cohorts, researchers examined 32,646 controls and 14,245 individuals with CHD, performing gene sequencing of LPL [38]. They identified 188 heterozygous carriers of damaging LPL mutations, including loss-of-function and missense variants. These rare mutations were significantly associated with elevated TG levels and an increased risk of CHD (Odds ratio 1.84; 95% CI 1.35–2.51; *p* < 0.001).

A GWAS found that 5% of the Amish population in Lancaster, Pennsylvania, carry a null mutation (R19X) in the APOC3 gene, which encodes ApoC-III [39]. This mutation is associated with lower fasting and postprandial TG and remnant cholesterol levels, as well as a reduced burden of coronary atherosclerosis. Carriers were significantly less likely than non-carriers to have detectable coronary artery calcium (CAC) (OR 0.35; 95% CI 0.21–0.60; *p* = 0.002) or CAC scores >100 Agatston units (OR 0.40; 95% CI 0.18–0.85, *p* = 0.01). These observations have been replicated in populations of European, African and Asian ancestry [40,41,42]. In a study of the Copenhagen population involving 75,725 participants, 10,797 developed ischemic vascular disease [40]. Heterozygosity for APOC3 loss-of-function mutations, compared to non-carriers, was associated with a mean reduction in non-fasting TG levels of 44% (*p* < 0.001) and a 41% reduction in ischemic vascular disease (*p* = 0.007). Another study found that heterozygosity for APOC3 loss-of-function mutations was associated with a 39% reduction in TG levels [41]. Among 498 carriers of any rare APOC3 mutation, the risk of CHD was 40% lower compared to 110,472 non-carriers. Finally, genetic analysis of over 10,000 participants from consanguineous families in Pakistan identified four individuals who were homozygous for an APOC3 loss-of-function mutation [42]. Compared to non-carriers, these homozygotes exhibited a 60% reduction in TG levels.

Whole-exome sequencing of two siblings presenting with combined hypolipidemia identified two distinct nonsense mutations in the ANGPTL3 gene [43]. In the DiscovEHR study, individuals with heterozygous loss-of-function variants in ANGPTL3 had significantly lower levels of TGs, HDL-C and LDL-C [44]. These genetic variants were associated with a 41% reduction in the odds of CHD among 53,532 participants. Data from 20,092 individuals in the Myocardial Infarction Genetics Consortium studies, including 60 heterozygous carriers of an ANGPTL3 loss-of-function mutation, showed that carriers had a 17% reduction in circulating TGs and a 12% reduction in LDL-c compared to non-carriers [45]. Carrier status was associated with a 34% lower risk of CHD.

Other angiopoietin-like proteins, including ANGPTL4, inhibit LPL and promote the elevation of TRLs. Loss-of-function variants in ANGPTL4 have also been associated with reduced TG levels and a lower risk of CHD in the DiscovEHR study [46].

Fibroblast growth factor 21 (FGF21), a stress-responsive hormone, participates in the regulation of lipid and glucose metabolism, by decreasing VLDL synthesis and facilitating TRL catabolism within adipose tissue [47]. Currently, no pathogenic loss-of-function or clinically relevant gain-of-function variants in FGF21 have been associated with any human disorder. However, several single SNPs within or near the FGF gene have been investigated for associations with diverse human phenotypes. A GWAS conducted using data from the UK Biobank and other GWAS consortia identified an association between the SNP rs838133 and reduced levels of TG, LDL-C and waist-to-hip ratio [48]. This FGF21 variant was strongly linked to lower venous thromboembolism risk and showed suggestive inverse associations with ASCVD. Another GWAS conducted in a Swedish cohort identified strong associations between specific variants in the FGF21 gene and circulating FGF21 levels, as well as an improved lipid profile [49].

### 3.3. Biological Data

TRLs, including VLDL and chylomicrons, are generally too large to penetrate the arterial intima. However, their cholesterol-rich remnant particles, formed through lipolysis by LPL and measuring 70 nm or smaller, can infiltrate the subendothelial space at sites of endothelial dysfunction and contribute to atherogenesis [26,50]. Furthermore, the amount of cholesterol carried per particle in remnant lipoproteins, partly due to cholesteryl ester transfer from HDL via CETP, is substantially greater than that transported by LDL [50]. Once retained within the arterial wall, TRLs can be phagocytosed by macrophages, contributing to foam cell formation, plaque development, and the progression of atherosclerosis.

Although TGs themselves are not directly linked to atherogenesis, free fatty acids released during delipidation of TRLs can trigger proinflammatory responses in endothelial cells and monocyte-derived macrophages [51]. This effect is mediated through the activation of toll-like receptors on myeloid cells of the innate immune system and is particularly associated with saturated fatty acids [52]. Unlike saturated fatty acids, polyunsaturated free fatty acids, such as omega-3s, exert anti-inflammatory effects rather than proinflammatory activity.

Free fatty acids released by the lipolytic action of LPL can also exert cytotoxic effects by promoting the generation of reactive oxygen species primarily by neutrophils [51]. The inflammatory response promotes activation of platelets and the coagulation system, as well as inhibition of endogenous antithrombotics, thereby establishing a link with thrombogenesis [53].

## 4. Management of Hypertriglyceridemia

The management of HTG involves a comprehensive approach that includes identifying and correcting secondary causes, instituting lifestyle modifications, and applying pharmacological strategies tailored to TG levels and cardiovascular (CV) risk. While extreme HTG poses a risk for pancreatitis, moderate elevations are primarily targeted for reducing ASCVD risk, though the benefit of TG-lowering therapies remains debated.

### 4.1. Identifying Secondary Causes

Evaluation for secondary contributors is a critical first step. As mentioned, common reversible causes include uncontrolled diabetes, excessive alcohol intake, hypothyroidism, nephrotic syndrome, and medication-related dyslipidemia [14]. Addressing these underlying conditions can substantially reduce TG levels and, in many cases, normalize them without the need for pharmacologic therapy.

### 4.2. Therapeutic Goals by Triglyceride Level

In severe HTG (TG ≥ 1000 mg/dL or ≥11.3 mmol/L), the immediate goal is to prevent acute pancreatitis, a potentially life-threatening complication. Observational studies suggest the risk of pancreatitis increases exponentially when TG levels exceed 1000 mg/dL, with some considering intervention at >500 mg/dL in high-risk individuals. Interventions include a very low-fat diet, insulin therapy if hyperglycemia is present, and in select cases, plasmapheresis [54].

In patients with TG levels 200–999 mg/dL (2.3–11.2 mmol/L), the focus shifts to managing ASCVD risk. Elevated TG in this range is often part of an atherogenic milieu which is commonly seen in metabolic syndrome and type 2 diabetes. However, the independent causal role of TGs in ASCVD, and the benefit of pharmacologically lowering them, remains an area of ongoing debate and investigation [55].

### 4.3. Lifestyle and Dietary Intervention

Lifestyle modification is foundational across the full spectrum of HTG. Nutritional interventions include reducing simple carbohydrates, added sugars, and alcohol, alongside increasing intake of omega-3–rich foods such as fatty fish [56]. Weight loss of 5–10% can significantly lower TG levels, especially in insulin-resistant individuals. Aerobic exercise (150–300 min/week) improves insulin sensitivity and enhances TG clearance. Referral to a registered dietitian is recommended for sustained behavioural change.

### 4.4. Pharmacologic Therapy

#### 4.4.1. Fibrates

Fibrates are PPAR-α agonists that lower TGs by 30–50% and modestly increase HDL-C. Their role in reducing cardiovascular events has been contentious. In the FIELD trial, fenofibrate failed to significantly reduce the primary CV endpoint in patients with type 2 diabetes, though subgroup analyses suggested benefit in those with high TG and low HDL-C [57]. Similarly, ACCORD-Lipid, which tested fenofibrate added to simvastatin in diabetic patients, showed no overall reduction in major CV events. However, a prespecified subgroup of patients with TG > 204 mg/dL and HDL-C < 34 mg/dL experienced a 31% relative risk reduction, suggesting benefit in those with pronounced atherogenic dyslipidemia [58].

The PROMINENT trial, published in 2022, tested pemafibrate, a novel selective PPAR-α modulator, in over 10,000 statin-treated patients with type 2 diabetes and mild to moderate HTG (median TG 271 mg/dL). Despite significant reductions in TGs (−26%) and remnant cholesterol, the trial did not demonstrate any reduction in CV events [59]. Notably, LDL-C rose slightly in the pemafibrate group, and levels of apoB—a key marker of atherogenic particle number—were unchanged. These findings suggest that pemafibrate, despite improving TG levels, may not reduce cardiovascular risk unless apoB-containing lipoproteins are also reduced. Whether this represents a class effect failure or a drug-specific issue remains a matter of debate.

#### 4.4.2. Omega-3 Fatty Acids

Omega-3 fatty acids reduce TG synthesis and promote clearance via enhanced β-oxidation. The clinical efficacy of omega-3s, however, varies significantly by formulation, dose, and study population.

Earlier studies, such as JELIS, an open-label trial in Japan using 1.8 g/day of eicosapentaenoic acid (EPA) added to low-dose statin, showed a 19% reduction in coronary events, though generalizability outside East Asia was questioned due to dietary and pharmacologic differences [60]. The ASCEND trial in diabetics tested a low-dose (1 g/day) omega-3 formulation in a primary prevention population and found no benefit, highlighting that both dose and patient selection are critical to efficacy [61].

The REDUCE-IT trial marked a turning point. In over 8000 statin-treated patients with elevated TG (135–499 mg/dL) and either established ASCVD or diabetes plus additional risk factors, high-dose EPA (4 g/day) led to a 25% relative risk reduction in major CV events [62]. Importantly, the benefit was not fully explained by TG reduction, suggesting additional anti-inflammatory, anti-thrombotic, or plaque-stabilizing effects of EPA. Some critics questioned the use of mineral oil as placebo in REDUCE-IT, which may have exaggerated treatment benefit. However, regulatory agencies including the U.S. Food and Drug Administration (FDA) and the European Medicines Agency accepted the results as valid.

In contrast, the STRENGTH trial tested a mixed EPA/ docosahexaenoic acid (DHA) formulation (omega-3 carboxylic acids, 4 g/day) in a similar high-risk population and found no CV benefit [63]. This discrepancy has fueled considerable attention, with particular attention on the placebo controversy, the use of mineral oil in REDUCE-IT (corn oil in STRENGTH), which may have adversely affected the control group. Other potential explanations include differences in the biological effects of DHA and varying levels of achieved plasma EPA. The failure of DHA-containing combinations to show benefit—also reflected in earlier trials—has focused attention on EPA-only formulations as the preferred option for cardiovascular risk reduction.

#### 4.4.3. Statins

Though not TG-specific agents, statins modestly reduce TG levels—especially in patients with elevated baseline TG—and are foundational for ASCVD risk reduction. Their benefits extend across TG levels and should remain first-line pharmacologic therapy in patients with mixed dyslipidemia, elevated LDL-C, non-HDL-C or ApoB levels. This is supported by consistent reductions in CV events across multiple statin trials, regardless of baseline TG [64].

### 4.5. Guideline Recommendations

The American guidelines recommend addressing secondary causes and lifestyle first. For patients with TG ≥ 500 mg/dL, the priority is pancreatitis prevention [65]; for those with TG 135–499 mg/dL and high ASCVD risk, icosapent ethyl may be considered as an adjunct to statin therapy [66]. The European guidelines emphasize targeting non-HDL-C and apoB in patients with elevated TG, reflecting the shift away from TG as a primary treatment target [67]. Notably, the American College of Cardiology expert consensus decision pathways offer more granular guidance on incorporating therapies like icosapent ethyl based on residual risk phenotyping [66].

### 4.6. Clinical Uncertainty

While elevated TGs are associated with ASCVD in observational studies, randomized controlled trials suggest that lowering TG alone is insufficient unless atherogenic lipoproteins (e.g., apoB-containing remnants) are also reduced. Mendelian randomization studies support a causal role for TRLs, but not necessarily for TGs themselves [55]. Implementation of ApoB and non-HDL-C measurement in clinical practice remains suboptimal, partly due to access, awareness, and uncertainty around treatment thresholds. Hence, current treatment strategies prioritize comprehensive lipid and risk factor management rather than targeting TG in isolation.

## 5. Emerging Therapies

Advancements in human genetics are increasingly guiding the development of novel targeted therapies [26,35]. This progress is further enabled by the availability of monoclonal antibodies and RNA-based technologies, including small interfering RNAs (siRNA) and antisense oligonucleotide (ASO). In this context, therapeutic strategies targeting TRLs have been developed and are currently undergoing clinical evaluation.

### 5.1. Apolipoprotein C-III Inhibitors

ApoC-III is a small peptide composed of 79 aminoacid residues, predominantly associated with TRLs [68]. It exerts pleiotropic effects across several pathways, including TRL metabolism, atherogenesis, inflammation, glucose homeostasis, and the development of cardiovascular and neurological disorders. ApoC-III is a well-established endogenous inhibitor of LPL and three compounds targeting ApoC-III synthesis have been developed.

Volanesorsen is a second-generation ASO that inhibits the synthesis of ApoC-III by targeting APOC3 mRNA [69,70,71]. Three Phase 3 clinical trials, including 220 patients with HTG, showed a reduction of −71.2% to −88% in fasting serum TG and −61% % to −84.2% in serum ApoC-III (Table 1) [72,73,74]. A meta-analysis of the three trials demonstrated a significant reduction in the incidence of acute pancreatitis over 6 to 12 months with volanesorsen 300 mg compared to placebo (1.7% vs. 10.5%; Odds Ratio 0.18; 95% CI 0.04 to 0.82) [75]. The most common adverse events in the volanesorsen groups were injection-site reactions and decreases in platelet counts. Volanesorsen is approved in several countries as an adjunct to diet in adults with FCS [76]. It has not been approved in the United States or Canada.

Olezarsen is a third-generation ASO developed to target APOC3 mRNA and inhibit the synthesis of ApoC-III. It is conjugated with N-acetylgalactosamine (GalNAc), which enables selective hepatic uptake via asialoglycoprotein receptors, leading to enhanced potency, reduced dosing requirements, and an extended therapeutic interval [77]. In a Phase 2 study, olezarsen demonstrated dose-dependent reductions in ApoC-III and TG levels (Table 1) [78]. Subcutaneous doses of 50 mg and 80 mg, administered monthly, were selected for further clinical evaluation [79,80]. In a Phase 3 study involving patients with genetically confirmed FCS, only the 80 mg dose of olezarsen significantly reduced TG levels compared to placebo. After 53 weeks, olezarsen treatment groups reported a single episode of acute pancreatitis, compared to 11 episodes in the placebo group (rate ratio 0.12; 95% CI 0.02 to 0.66) and no episodes of thrombocytopenia in all groups. Platelet abnormalities were comparable between the olezarsen and placebo groups [78,79,80]. Four Phase 3 clinical trials are ongoing to evaluate the effect of olezarsen on the percent change in fasting TG levels compared to placebo at 53 weeks. These studies also evaluate whether TG lowering can reduce hepatic fat, as measured by magnetic resonance imaging (NCT05079919 and NCT05552326), and coronary plaque progression, as assessed by coronary computed tomographic angiography (NCT05610280) [81]. The FDA approved olezarsen, used with diet, as the first-ever treatment to reduce TG in adults with FCS.

Plozasiran is a siRNA designed to inhibit the synthesis of ApoC-III. In Phase 2b studies, in patients with mixed hyperlipidemia or severe HTG, plozasiran showed a dose-related reduction in ApoC-III and TG concentrations (Table 1) [82,83]. The Phase 3 PALISADE trial enrolled 75 patients with FCS across three treatment arms [84]. At 10 months, plozasiran reduced TG levels by up to 80% compared to placebo. Additionally, the incidence of acute pancreatitis was significantly lower in the plozasiran groups, with an odds ratio of 0.17 (95% CI 0.03 to 0.94) relative to placebo. In patients with diabetes or prediabetes receiving plozasiran, a mild increase in glycated hemoglobin levels was observed. Elevated hepatic transaminases, up to three times the upper limit of normal, were also reported in some patients treated with plozasiran. Three Phase 3 clinical trials in patients with severe HTG (≥500 mg/dL [5.65 mmol/L]; SHASTA-3 [NCT06347003 and SHASTA-4 [NCT06347016]) or with HTG (≥150 mg/dL [≥1.69 mm/L] and ≤499 mg/dL [≤5.64 mmol/L]; MUIR-3 [NCT06347133]) are ongoing.

**Table 1:** Phase 2 and Phase 3 clinical trials investigating ApoC-III inhibitors.

Clinical Trial	Population	Design	Active Treatment	Control	Duration (Months)	TG Reduction (%)	RC Reduction (%)	LDL-C Reduction (%)	ApoC-III Reduction (%)
**Volanesorsen**									
**NCT01647308 [70]** Phase 2	N = 15 TG > 200 and <500 mg/dL and type 2 diabetes	Double-blind 2:1	Volanesorsen 300 mg q1week	Placebo	12 weeks	−69.1 at 3 months	−75.5 at 3 months	−3.2 at 3 months	−87.5 at 3 months
**NCT01529424 [71]** Phase 2	N = 85 HTG of 350 to 2000 mg/dL (or 225–2000 mg/dL if on fibrate therapy)	Double-blind, dose ranging (1 of 3 doses or placebo)	Volanesorsen 100 mg q1week 200 mg q1week 300 mg q1week	Placebo	13 weeks	−31.3 to −70.9 at 3 months	−54.1 to −86.7 at 3 months	+48.0 to +118.3 at 3 months	−40.0 to −79.6 at 3 months
**COMPASS [72]** **NCT02300233** Phase 3	N = 114; MH or FCS and TG ≥ 500 mg/dL	Double-blind 2:1	Volanesorsen 300 mg q1week	Placebo	26 weeks	−71.2 at 3 months	−72.4 at 3 months	+95.5 at 3 months	−76.1 at 3 months
**APPROACH [73]** **NCT02211209** Phase 3	N = 66; FCS and TG ≥ 750 mg/dL	Double-blind 1:1	Volanesorsen 300 mg q1week	Placebo	52 weeks	−76.5 at 3 months	−71.7 at 3 months	+135.6 at 3 months	−84.2 at 3 months
**BROADEN [74]** **NCT02527343** Phase 3	N = 40; FPLD and TG ≥ 500 mg/dL (≥200 mg/dL if genetic diagnosis/family history), type 2 diabetes and fatty liver	Double-blind 1:1	Volanesorsen 300 mg q1week	Placebo	52 weeks	−88.0 at 3 months	-	-	−61.0 at 3 months
**Olezarsen**									
**NCT03385239 [78]** Phase 2	N = 114 TG 200–499 mg/dL and established ASCVD or high ASCVD risk	Double-blind dose ranging (1 of 4 doses or placebo)	Olezarsen 10 mg q4week 15 mg q2week 10 mg q1week 50 mg q4week	Placebo	52 weeks	−23.0 to −60.0 at 6 months	−22.0 −67.1 at 6 months	−6.0 to +16.0 at 6 months	−29.0 to −74.0 at 6 months
**Bridge-TIMI 73a [79]** **NCT05355402** Phase 2b	N = 154 TG 150–499 mg/dL and elevated ASCVD risk or severe HTG (TG > 500 mg/dL)	Double-blind 3:1	Olezarsen 50 mg q4week 80 mg q4week	Placebo	49 weeks	−60.5 and −67.6 at 6 months	−60.0 and −68.0 at 6 months	+5.9 and +13.0 at 6 months	−65.6 and −76.4 at 6 months
**BALANCE [80]** **NCT04568434** Phase 3	N = 66 FCS and TG ≥ 880 mg/dL	Double-blind 3:1	Olezarsen 50 mg q4week 80 mg q4week	Placebo	49 weeks	−22.4 and −43.5 at 6 months	−22.4 and −43.5 at 6 months	+34.7 and 64.9 at 6 months	−57.9 and −66.1 at 6 months
**Plozasiran**									
**MUIR [82]** **NCT04998201** Phase 2b	N = 353 TG 150–499 mg/dL with LDL ≥ 70 mg/dL or non-HDL ≥ 100 mg/dL	Double-blind dose ranging (1 of 4 doses or placebo)	Plozasiran 10 mg q12week; 25 mg q12week; 50 mg q12week; 50 mg q24week	Placebo	48 weeks	−44.2 to −62.4 at 24 weeks	−44.2 to −62.4 at 24 weeks	−41.7 to −54.2 at 24 weeks	−10.4 to +6.0 at 24 weeks
**SHASTA-2 [83]** **NCT04720534** Phase 2b	N= 229; Severe HTG (≥500 mg/dL)	Double blind, dose ranging (1 of 3 doses or placebo)	Plozasiran 10 mg q12week 25 mg q12week 50 mg q12week	Placebo	48 weeks	−48.8 to −57 at 24 weeks	−58.8 to −63.6 at 24 weeks	+25.8 to +60.3 at 24 weeks	−67.7 to −77.4 at 24 weeks
**PALISADE [84]** **NCT05089084** Phase 3	N = 75 FCS with TG ≥ 880 mg/dL, low LPL activity or a history of acute pancreatitis	Double-blind 2:1	Plozasiran 25 mg q3month 50 mg q3month	Placebo	12 months	−80.0 and −78.0 at 10 months	−60.1 and −49.6 at 10 months	+105.9 and +83.0 at 10 months	−93.0 and −96.0 at 10 months

ASCVD: Atherosclerotic cardiovascular disease; FCS: Familial chylomicronaemia syndrome; FPLD: Familial partial lipodystrophy; HDL-C: High-density lipoprotein cholesterol; HTG: Hypertriglyceridemia; LDL-C: Low-density lipoprotein cholesterol; LPL: Lipoprotein lipase; MH: Multifactorial Hypertriglyceridemia; RC: Remnant cholesterol; TG: Triglyceride.

### 5.2. Angiopoietin-like 3 Inhibitors

ANGPTL3 belongs to a family of eight angiopoietin-like proteins and is produced and secreted by the liver [85,86]. ANGPTL3 is predominantly active after feeding, promoting the inhibition of LPL activity in oxidative tissues and thereby allowing the replenishment of white adipose tissue depots.

Vupanorsen is a second-generation ASO conjugated with GalNAc designed to target ANGPTL3 mRNA [87]. In a Phase 2 dose-ranging clinical trial involving 105 patients with TG levels >150 mg/dL (1.7 mmol/L), type-2 diabetes, and hepatic steatosis, vupanorsen 80 mg resulted in a 53% reduction in TG and a 59% reduction in ANGPTL3 levels (Table 2) [87]. Approximately 20% of patients treated with vupanorsen reported injection-site reactions. In the Phase2b trial TRANSLATE-TIMI 70 with 286 patients, vupanorsen decreased TG and ANGPTL3 levels in a dose-dependent manner [88]. Vupanorsen was associated with a dose-dependent increase in the incidence of injection-site reactions, elevations of hepatic transaminases exceeding three times the upper limit of normal and increases in hepatic fat fraction. Due to this unfavourable safety profile, the clinical development of vupanorsen was discontinued.

Zodasiran is a siRNA conjugated with GalNAc that inhibits the synthesis of ANGPTL3. In the Phase 2 dose-ranging ARCHES-2 clinical trial involving 204 patients with mixed hyperlipidemia, zodasiran reduced TG levels up to 63% and ANGPLT3 up to 73% (Table 2) [89]. Zodasiran treatment resulted in a reduction in liver fat content, with the 200 mg dose achieving up to a 28% decrease. Apart from a transient increase in glycated hemoglobin levels observed in patients with preexisting diabetes who received the highest dose of zodasiran, adverse event rates were similar between the treatment and placebo groups. No significant decreases in platelet count or elevations in transaminases were reported. Despite these results, Arrowhead Pharmaceuticals discontinued zodasiran in favour of the ApoC-III inhibitor plozasiran.

Solbinsiran is a GalNAc-conjugated siRNA that inhibit the hepatic synthesis of ANGPTL3 [90]. Solbinsiran was evaluated in the Phase 2b PROLONG-ANG3 clinical trial, which enrolled 205 patients with mixed dyslipidemia and TG levels ranging from 150 and 500 mg/dL (Table 2). At day 180, treatment with the 800 mg dose of solbinsiran resulted in a 52.5% reduction in TG levels and a 76.6% reduction in ANGPTL3 levels. The therapy was well tolerated, with a low incidence of adverse events. Phase 2 findings demonstrate that solbinsiran is both effective and well tolerated, supporting its progression into Phase 3 evaluation.

Evinacumab is a fully human monoclonal antibody that binds ANGPTL3 with high affinity [91]. Evinacumab was initiated in clinical development for patients with hypercholesterolemia (Table 2) [92,93]. In a Phase 2 study enrolling 272 patients with refractory disease, treatment with evinacumab led to reductions in LDL-C of up to 50% and in TG by over 50% [92]. In 65 patients with homozygous familial hypercholesterolemia included in the ELIPSE clinical trial, evinacumab achieved a 41% reduction in LDL-C and a 55% reduction in TG levels [93]. Adverse event rates were similar in patients receiving evinacumab and those receiving placebo. Evinacumab has received approval in the United States for the treatment of homozygous familial hypercholesterolemia in patients aged 5 years and older, and in Europe for patients aged 6 months and older. In a Phase 2 clinical trial involving patients with severe HTG, evinacumab did not reduce TG levels in patients with FCS [94]. However, a TG-lowering benefit was observed in patients with MH.

**Table 2:** Phase 2 and Phase 3 clinical trials investigating ANGPTL3 inhibitors.

Clinical Trial	Population	Design	Active Treatment	Control	Duration (Months)	TG Reduction (%)	RC Reduction (%)	LDL-C Reduction (%)	ANGPTL3 Reduction (%)
**Vupanorsen**									
**NCT03360747 [87]** Phase 2	N = 105 TG > 150 mg/dL, type 2 diabetes and hepatic steatosis	Double-blind	Vupanorsen 40 mg q4week 80 mg q4week 20 mg q1week	Placebo	6 months	−36 to −53 at 6 months	−35 to −47 at 6 months	+6 to −12 at 6 months	−41 to −59 at 6 months
**TRANSLATE-TIMI 70 [88]** **NCT04516291** Phase 2b	N = 286 Statin-treated with non–HDL-C ≥ 100 mg/dL and TG 150–500 mg/dL	Double-blind, dose ranging (1 of 7 dose regimens or placebo)	Vupanorsen 80, 120, or 160 mg q4 weeks or 60, 80, 120, or 160 mg q2weeks	Placebo	24 weeks	−41.3 to −56.8 at 24 weeks	-	−9.1 to −17.3 at 24 weeks	−56.6 to −81.9 at 24 weeks
**Zodasiran**									
**ARCHES-2 [89]** **NCT04832971** Phase 2	N = 204 Mixed hyperlipidemia with TG 150–499 mg/dL, LDL-C ≥ 70 mg/dL or non-HDL-C ≥ 100 mg/dL	Double-blind, dose ranging (1 of 3 doses or placebo)	Zodasiran 50 mg q12week; 100 mg q12week; 200 mg q12week	Placebo	6 months	−51.2 to −63.1 at 6 months	−53.0 to −62.0 at 6 months	−12.0 to −18.0 at 6 months	−54.3 to −73.7 at 6 months
**Solbinsiran**									
**PROLONG-ANG3 [90]** **NCT05256654** Phase 2b	N = 205 Mixed dyslipidemia: TG 150–499 mg/dL), LDL > 70 mg/dL, non-HDL > 130 mg/dL; on statin for >2 months	Double-blind, dose ranging (1 of 3 doses or placebo)	Solbinsiran 100 mg D0/D90 400 mg D0/D90 800 mg D0/D90	Placebo	270 days	−36.3 to −52.5 at day 180	-	−1.3 to −16.8 at day 180	−54.3 to −76.6 at day 180
**Evinacumab**									
**NCT03175367 [92]** Phase 2	N = 272 Hypercholesterolemia with or without ASCVD, refractory to maximum OMT (statin/PCSK9i)	Double-blind, dose ranging (SC or IV)	Evinacumab (SC) 300 mg q2week 300 mg q1week 450 mg q1week (IV) 15mg/Kg q4weeks 5mg/Kg q4weeks	Placebo	16 weeks	−38 to −53.4 at 16 weeks	-	−29.7 to −49.9 at 16 weeks	-
**ELIPSE HoFH [93]** **NCT03399786** Phase 3	N = 65 HoFH under LLT (LDL > 70 mg/dL)	Double-blind, 2:1	Evinacumab (IV) 15mg/Kg q4weeks	Placebo	24 weeks	−55.0 at 24 weeks	−79.6 at 24 weeks	−47.1 at 24 weeks	-
**NCT03452228 [94]** Phase 2	N = 51 Severe HTG (≥500 mg/dL); FCS or MH with or without LPL mutations	Double-blind, 2:1	Evinacumab (IV) 15mg/Kg q4weeks	Placebo	12 weeks	FCS: −27.7; MH: −64.8 to −81.7 at 12 weeks	FCS: −37.5; MH: −62.8 to −79 at 12 weeks	FCS: +25; MH: +26.5 to +32 at 12 weeks	-

ASCVD: Atherosclerotic cardiovascular disease; FCS: Familial chylomicronaemia syndrome; HDL-C: High-density lipoprotein cholesterol; HoFH: Homozigous Familial Hypercholesterolemia; HTG: Hypertriglyceridemia; LDL-C: Low-density lipoprotein cholesterol; LLT: Lipid-lowering therapy; LPL: Lipoprotein lipase; MH: Multifactorial Hypertriglyceridemia; OMT: Optimal medical therapy; PCSK9i: Proprotein convertase subtilisin/kexin type 9; RC: Remnant cholesterol; TG: Triglyceride.

### 5.3. Other Therapeutic Strategies

Agents targeting inhibition of ANGPTL4 are under clinical development. MAR001 is a humanized monoclonal antibody administered subcutaneously that inhibits ANGPTL4 (NCT05896254) [95]. A GalNAc-conjugated ASO is currently under evaluation in a Phase 2 clinical trial [96].

Pegozafermin is an FGF21 analog developed using glycoPEGylation technology, which extends its half-life and reduces off-target FGF21 effects [97]. In the Phase 2 ENTRIGUE clinical trial (NCT04541186) involving 85 patients with TG levels between 500 mg/dL and 2000 mg/dL, pegozafermin administered subcutaneously demonstrated a dose-related reduction in TG levels [98]. Additionally, hepatic fat fraction was reduced by 42.2%. A Phase 3 clinical trial (ENTRUST NCT05852431) evaluating pegozafermin in patients with severe HTG is currently in preparation.

## 6. Conclusions and Future Directions

HTG is a common multifactorial metabolic disorder, often influenced by genetic susceptibility and driven by abnormalities in lipid metabolism. Evidence from epidemiology, genetics and biology implicates TRLs, but not necessarily TGs themselves, as causal in ASCVD. Severe HTG is also linked to pancreatitis and hepatic steatosis. Current pharmacologic therapies exhibit notable limitations in effectively managing severe HTG and in reducing the risk of ASCVD.

Emerging therapies targeting TRLs metabolism showed promising results in phase 2 and small phase 3 trials. ApoC-III inhibitors effectively reduce TG levels, including in severe HTG such as FCS, through both LPL-dependent and independent mechanisms. ANGPTL3 inhibitors demonstrate promise in mixed hyperlipidemia by exerting favourable effects across the full range of atherogenic lipoproteins. These therapies mark a paradigm shift by targeting TRL remnants, not just TGs, to reduce ASCVD risk. Other therapeutic approaches, including ANGPTL4 inhibitors and FGF21 analogs, are currently in early-stage clinical development.

Given the rapid progress in this field, effective therapies targeting TRLs and HTG may soon become available. However, definitive evidence from large randomized cardiovascular outcome trials remains essential to establish the clinical benefit of these emerging therapies.
